# Recent developments in psychosocial interventions for borderline personality disorder

**DOI:** 10.12688/f1000research.18561.1

**Published:** 2019-04-26

**Authors:** Christina M Temes, Mary C Zanarini

**Affiliations:** 1 McLean Hospital, Belmont, MA, USA; 2 Harvard Medical School, Boston, MA, USA

**Keywords:** borderline personality disorder, treatment, intervention, psychotherapy

## Abstract

Borderline personality disorder (BPD) is a serious psychiatric disorder that affects multiple symptomatic domains and is associated with an increased risk of suicidality. Several empirically supported treatments for BPD have been developed in recent years for adults with BPD. More recent work has focused on tailoring or applying (or both) these existing treatments to specific patient populations, including patients with certain types of comorbidity (for example, BPD and post-traumatic stress disorder or antisocial personality disorder) and younger patients. Other work has involved developing treatments and models of treatment delivery that address concerns related to access of care. Relatedly, new adjunctive and technology-assisted interventions have been developed, adding to the growing repertoire of treatment options for these patients. Advances in the last several years address specific treatment needs and offer cost-efficient options for this diverse patient population.

Borderline personality disorder (BPD) is a serious mental illness that is characterized by difficulties in affect regulation, interpersonal relationships, and impulsivity and is associated with an elevated risk of suicidal behavior ^1, 2^. A number of specialized treatments, including dialectical behavior therapy (DBT), mentalization-based therapy (MBT), and transference-focused psychotherapy (TFP), have been developed over the past few decades and have been found to be similarly efficacious in recent meta-analytic work ^3^. Within the past few years, additional interventions for BPD have been developed, and specific foci are targeting comorbidity common to patients with BPD, improving access to care, tailoring treatments to younger patients, and developing adjunctive or web-based interventions to further help these patients in a cost-efficient way.

## Treatment of borderline personality disorder and comorbid conditions

Several newly developed and evaluated psychosocial treatments for BPD include adaptations of existing treatments to target both BPD and commonly co-occurring conditions. These treatments include two adaptations of DBT for patients with BPD (or BPD features) and comorbid post-traumatic stress disorder (PTSD) ^4, 5^. Two other recent studies have examined the efficacy of two existing treatments for BPD—MBT and Systems Training for Emotional Predictability and Problem Solving (STEPPS)—for treating BPD and comorbid antisocial personality disorder (ASPD) ^6, 7^. Additionally, there is emerging evidence that using a transdiagnostic approach to treatment may be beneficial for patients with BPD and mood/anxiety disorders ^8^.

With respect to the treatment of BPD with comorbid PTSD, two treatment approaches using DBT have been tested recently. Harned *et al*. ^4, 9^ developed a treatment that combines standard DBT with prolonged exposure (PE), an evidence-based treatment for PTSD ^10^. An initial study of this treatment, named DBT-PE ^4^, found that patients with comorbid BPD, PTSD, and acute suicidality or self-injury who received DBT-PE had higher rates of remission from PTSD than patients who received DBT alone (that is, 80% versus 40%). Patients who completed DBT-PE, compared with standard DBT, also exhibited improvements in other symptomatic domains (that is, dissociation, depression, anxiety, guilt, shame, and global symptom severity) of moderate to large effect sizes. Additionally, across both treatments, improvements in PTSD symptoms and post-traumatic cognitions predicted improvements on multiple functional domains ^9^, suggesting a potential mechanism through which this treatment operates.

Another modification of DBT, developed by Bohus *et al*. ^5^, was designed for patients experiencing PTSD following childhood sexual abuse as well as difficulties with emotion dysregulation (including BPD). This modular, 12-week residential treatment combines elements of DBT with other trauma-focused exposure and cognitive approaches ^10, 11^. Patients who received DBT-PTSD, compared with treatment as usual, showed greater improvements in PTSD symptomatology, global social functioning, and depression. They also seemed to tolerate the treatment well. (That is, they did not have increases in self-destructive behaviors or PTSD symptoms and so on.) Additionally, level of initial BPD symptomatology was not a predictor of treatment course.

Researchers have also adapted existing psychotherapies with empirical support for BPD to treat comorbid personality disorders, particularly ASPD. One such treatment has been an adaptation of MBT for patients with BPD and comorbid ASPD ^6^. MBT aims to help patients better understand their own and others’ mental states and intentions. The developers expected that engaging in this kind of active thinking about others’ intentions rather than acting impulsively on the basis of initial instincts could reduce violence and other antisocial behaviors. Results from one investigation supported this idea, finding that patients with BPD plus ASPD treated with MBT showed greater improvements in ASPD-related behaviors than patients receiving similar outpatient treatment without MBT. These patients also showed reductions in anger, hostility, paranoia, self-harm, and suicide attempts as well as improvements in mood, general psychiatric symptoms, and interpersonal/social functioning when compared with patients receiving care as usual.

Another form of treatment that has been adapted to treat BPD comorbid with ASPD is STEPPS, an evidence-based adjunctive group treatment for BPD ^7^. This treatment includes components of psychoeducation, cognitive-behavioral therapy, and skills training ^12^. With respect to comorbid ASPD, two recently conducted studies (one conducted at an academic medical center and one at a correctional setting) demonstrated that patients with BPD plus ASPD who received STEPPS (compared with BPD alone) showed greater improvement on a variety of domains, including BPD symptomatology, general psychiatric symptoms, impulsiveness, positive affect, and positive/negative behaviors. These findings, though somewhat counterintuitive, suggest that patients with ASPD can benefit from evidence-based treatments and should not be excluded from these programs.

Another new treatment uses an established transdiagnostic approach to treat BPD. This treatment involves applying Barlow’s Unified Protocol for the Transdiagnostic Treatment of Emotional Disorders ^13^ for patients with BPD ^8^. This treatment is generally 16 to 20 outpatient sessions and is focused on the core negative emotional processes that underlie BPD and other conditions (for example, mood and anxiety disorders). A preliminary study by Sauer-Zavala *et al*. ^8^, which involved a clinical replication series of five patients with BPD and comorbid mood and anxiety disorders, yielded promising results, finding that most patients experienced clinically significant reductions in symptoms of BPD, depression, and anxiety as well as improvements in emotion regulation skills.

## Improving access to care

Other recent developments have focused on how to improve access to care for patients with BPD, including addressing long wait times for treatment in the community. Laporte *et al*. ^14^ and Paris ^15^ proposed and evaluated a “stepped care” model for treating BPD, which rests on the idea that some patients—particularly those with disorders for which outcomes are variable—may benefit from briefer interventions but that other patients may require more intensive treatment. Laporte *et al*. note that there is already evidence that patients with BPD can benefit from short-term (ST) versions of evidence-based treatments ^16, 17^. In their study, patients were offered an initial “step” of ST treatment, which consisted of 12 weeks of integrative therapy delivered in individual and group modalities. If patients who received ST did not improve or needed additional treatment, they were referred to an extended care (EC) clinic after a waiting period. Patients received EC, which consisted of two types of group therapy (one similar to ST and one based on group process), individual therapy, and psychopharmacology; treatment lasted for up to 2 years. Patients who attended the ST clinic exhibited significant declines in major BPD symptom areas (for example, depression, impulsivity, self-esteem, emotion dysregulation, self-harm, and suicidality), suggesting that brief treatment may be viable for many patients with BPD. Patients who continued on in EC also showed significant declines in these symptom areas.

In addition to the stepped-care model, treatment approaches have been described that can be incorporated into the practice of community practitioners with little deviation from their typical work. One such treatment is general psychiatric management (GPM) ^18^, which integrates evidence-based principles for treating BPD into a treatment that includes psychotherapy, case management, and symptom-targeted pharmacotherapy. GPM was originally tested as a condition in a large, randomly controlled trial in which it was compared with DBT ^19^. In this study, patients were randomly assigned to either 1 year of GPM or 1 year of DBT. GPM performed as well as DBT, and the two groups showed similar levels of improvement in suicidal and self-injurious behavior, BPD symptoms, and level of health-care utilization. Additionally, these gains were largely sustained at 2-year follow-up ^20^.

## Adjunctive and web-based interventions

In addition to standard psychotherapies for BPD and related conditions, some adjunctive treatments and technologically based interventions have been developed recently to help patients with BPD. These include two group-based adjunctive treatments with specific foci as well as web-based interventions.

Gratz *et al*. ^21^ developed a 14-week group therapy, called emotion regulation group therapy (ERGT), which was designed to be an adjunctive treatment for patients with BPD and recurrent deliberate self-harm (DSH). An initial evaluation of this treatment found that women with BPD and recurrent DSH who participated in ERGT show improvements on DSH, other self-destructive behaviors, emotion dysregulation, BPD symptomatology, depression, and quality of life post-treatment, and additional improvements were observed by 9-month follow-up. Changes in symptoms and problematic behaviors appear to be mediated by changes in emotion regulation ^22^, lending support to the proposed mechanism. Furthermore, patients who exhibited greater severity in multiple relevant domains as well as greater levels of comorbidity showed a greater response to treatment, suggesting that the type of patient who is likely to be referred to ERGT is also likely to benefit from it ^23^.

A different adjunctive treatment module was developed to enhance patients’ strengths. Specifically, Sandage *et al*. ^24^ developed a module based on forgiveness to be added to outpatient DBT. In one study, participants who completed this module showed improvements in forgiveness and decreases in psychiatric symptoms and attachment insecurity during the module and up to 6 weeks after. Similar changes were not observed following the preceding module of distress tolerance.

A newly developed internet-based psychoeducation program, developed by Zanarini *et al*. ^25^, has also been shown to be beneficial for patients with BPD in a randomized controlled trial. This program included regular web-based assessments and the completion of an online curriculum about BPD. During the first 12 weeks of the study, patients who completed the psychoeducation program showed greater improvement in impulsivity and overall functioning when compared with participants who did not complete the program. During the maintenance phase of the trial (months 6, 9, and 12), those who had been randomly assigned to read the curriculum showed greater improvements in all BPD symptom domains and overall severity of BPD symptoms compared with those who did not. Overall, these findings suggest that web-based psychoeducation is a promising and cost-efficient early intervention or adjunctive treatment for those newly diagnosed with BPD.

Other web-based interventions, including the REVISIT trial, a web-based adjunctive treatment based on schema therapy ^26^, are currently being tested. This intervention will combine elements of psychoeducation with self-paced interventions/exercises and is intended for patients who are also receiving care as usual.

## Treatment for adolescents and young adults

There has also been growing attention regarding earlier treatment of BPD, particularly in adolescence. There is ample evidence to suggest that the diagnosis of BPD is valid in adolescence ^27^ and that symptomatology during this developmental period is qualitatively different from experiencing a tumultuous period of normal development. In a recent commentary, Chanen and Thompson ^28^ stressed the importance of intervention in early stages of the disorder and advocated for a “clinical staging” approach in treating BPD in younger populations, like the stepped-care plan that has been evaluated in adults ^14, 15^: starting with simpler and more benign interventions in early stages of the disorder and increasing intensity if the disorder progresses.

Some full-package treatments initially developed and evaluated in adults with BPD have been adapted for adolescents with BPD. For instance, an adolescent form of DBT (DBT-A) ^29^ has been developed for adolescents with repeated self-harm and suicidality. This treatment follows a format similar to that of standard DBT, and individual therapy is combined with skills group and skills coaching; however, families are included in skills groups, and there is a separate family therapy component. In one trial ^29^, DBT-A was found to be superior to enhanced usual care in reducing self-harm, suicidal ideation, and depressive symptoms. Additionally, an adolescent form of MBT (MBT-A) ^30^ was developed and tested for adolescents with self-harm. Like DBT-A, MBT-A is similar in approach to standard MBT but also includes a monthly family therapy component and has an explicit focus on impulsivity and affect regulation. Compared with treatment as usual, MBT-A was superior in reducing self-harm and depression ^30^. Improved mentalization and reduced attachment avoidance appeared to be key mediating factors.

There are also multiple treatments that are in development and being tested to treat BPD in adolescents. These include the MOBY trial (principal investigator: Andrew Chanen) ^31^, which seeks to compare the efficacy of three types of early intervention models for adolescents and young adults with BPD: (1) the Helping Young People Early (HYPE) service model plus cognitive analytic therapy, (2) the HYPE service plus a control psychotherapy condition, and (3) a general youth mental health-care model plus control psychotherapy condition. Another ongoing trial (principal investigator: Emma Beck) ^32^ is testing a group-based MBT for adolescents.

## Summary and discussion

Recent developments in interventions for BPD address specific needs for the treatment of this population. Specifically, given that comorbidities are common in these patients ^33^, treatments that are developed with common comorbidities in mind (for example, DBT-PE and DBT-PTSD) or the evaluation of empirically supported treatments in patients with serious comorbidities (for example, MBT and STEPPS for ASPD plus BPD) represent important advances in BPD treatments. Specifically, these studies suggest that, despite comorbidities, patients benefit from these treatments and should be viewed as good candidates for these therapies. That being said, many full-package treatments are costly in time and money, and patients may benefit from alternative models of treatment delivery or adjunctive treatments with specific targets. Thus, the further development of staged treatment models, adjunctive treatments, and technologically based interventions ^14– 26^ may be particularly useful when cost-efficient interventions are needed, patients are newly diagnosed or awaiting full-package treatment (or both), or a specific deficit needs to be addressed in the context of an ongoing treatment. Additionally, given the growing evidence that BPD is a valid diagnosis in adolescence, treatments oriented at younger populations ^28– 32^ constitute a necessary and important advance.
